# Adolescent human immunodeficiency virus self-management: Associations with treatment adherence, viral suppression, sexual risk behaviours and health-related quality of life

**DOI:** 10.4102/sajhivmed.v21i1.1054

**Published:** 2020-04-29

**Authors:** Talitha Crowley, Anita van der Merwe, Martin Kidd, Donald Skinner

**Affiliations:** 1 Department of Nursing and Midwifery, Faculty of Medicine and Health Sciences, Stellenbosch University, Cape Town, South Africa; 2 Centre for Statistical Consultation, Stellenbosch University, Cape Town, South Africa; 3 Department of Public Health, Faculty of Medicine and Health Sciences, Stellenbosch University, Cape Town, South Africa

**Keywords:** HIV, adolescents, self-management, quality of life, antiretroviral treatment

## Abstract

**Background:**

With the advent of access to antiretroviral treatment (ART), human immunodeficiency virus (HIV) has become a chronic disease and self-management is an important component of its care. Research to date has not explored associations between adolescent HIV self-management and treatment adherence, viral suppression, sexual risk behaviour and health-related quality of life (HRQoL).

**Objectives:**

To explore the associations between adolescent HIV self-management and treatment adherence, viral suppression, sexual risk behaviour and HRQoL.

**Methods:**

A quantitative cross-sectional study of 385 adolescents living with HIV (ALHIV) aged 13–18 years, who were recruited from 11 healthcare facilities between March and August 2017 in the Cape Metropole of the Western Cape, South Africa, provided the data that were examined in this self-completed questionnaire. Validated scales were used to measure key variables. The most recent viral load (VL) was obtained from the participants’ clinic folder, taking into account that VL is done annually.

**Results:**

Adolescents who reported higher HIV self-management were more likely to be adherent to treatment (*t* = 4.435 [336], *p* < 0.01), virally suppressed (*t* = 2.376 [305], *p* = 0.02) and to practise consistent condom use (*t* = 1.947 [95], *p* = 0.54). Structural equation modelling (SEM) indicated a significant relationship between self-management and HRQoL (*r* = 0.43, *p* < 0.01), whilst non-adherent treatment taking behaviour, correlated with elevated VL log values. No significant correlation was found between self-management and sexual risk behaviour.

**Conclusion:**

Targeting adolescents’ skills related to HIV self-management in the clinical setting may improve adolescents’ treatment taking behaviour, viral suppression rates and their HRQoL.

## Background

Adolescents living with HIV (ALHIV) represent a growing proportion of the global population of people living with HIV. In 2018, 1 600 000 adolescents [1 100 000; 2 300 000] between the ages of 10 and 19 years were living with HIV. That year, 190 000 were newly infected.^1^ Sub-Saharan Africa (SSA) has the highest burden of HIV: 89% of the world’s ALHIV reside in this region. Of South Africa’s (SA) estimated 460 000 ALHIV, 52 000 new infections and 5 600 AIDS-related deaths were reported in 2018.^1^

Adolescents living with HIV can be divided into two groups: perinatally infected adolescents who are diagnosed as infants or children; and behaviourally/horizontally infected adolescents who likely acquired HIV through sexual transmission.^2^ One South African study reported 25.4% (*n* = 269) out of a sample of 1059 adolescents aged 10–19 years acquired HIV horizontally.^3^ Perinatally infected adolescents are usually treatment-experienced and more likely to suffer from the chronic effects of HIV infection such as delayed growth and development.^4^ Although the healthcare needs of perinatally and behaviourally infected adolescents may differ, shared healthcare concerns include medication non-adherence, risky sexual behaviour, psychosocial stressors and comorbid psychiatric illness.^2,5^

Adolescence is a complex developmental phase characterised by physical changes, cognitive and emotional advancement, sexual awakening and an increased sensitivity to relationships with peers.^6^ Adolescents have a need for autonomy and independence and ALHIV can be expected to begin to take responsibility for their care in preparation for transitioning from paediatric to adult care.^2^ Although cognitive ability and decision-making capacity have improved, adolescents remain vulnerable, that is, are preoccupied with social acceptance, may engage in risk-taking behaviour and the need to fit in with peers.^6^ Engagement in HIV care is further threatened by the perceived incongruence between HIV treatment and social goals.^7^ Deficits in cognitive function, memory and mental processing because of incompletely controlled HIV infection of the nervous system^4^ may further impair the self-management of ALHIV. Compared to adults, ALHIV have worse treatment outcomes.^8^ Adolescents living with HIV are more likely to be non-adherent or default their treatment. Evidence in support of specific approaches to the improved adherence of ALHIV is limited.^5,9,10,11^ Adolescents living with HIV require a differentiated care approach in clinical settings.^5^ In this regard, self-management is person-centred, an approach that may assist the adolescent to manage normal developmental tasks and to cope better with their HIV status,^12^ that is, with stigma, sexual health and behaviour, and emotional well-being.^5,13^

Self-management has been defined as a process by which individuals and families use knowledge and beliefs, self-regulate skills, abilities and social facilitation, to achieve health-related outcomes (Sawin, 2017:171).^7^ The Individual and Family Self-Management Theory (IFSMT) describes self-management as occurring in the context of various condition-specific, individual and environmental factors. The proximal outcome of self-management is behaviours such as engagement in treatment regimens (adherence). Distal outcomes include, for example, health status and health-related quality of life (HRQoL).^7^ Health-related quality of life includes perceived physical, emotional, mental, social and behavioural components of well-being and functioning.^14^

Adolescents living with HIV need skill to self-manage an array of challenges. These include being adherent to treatment (the medical management of their illness) as well as coping with HIV and stigma, namely, role and emotion management.^15,16,17^ Key self-management skills also include problem-solving, goal-setting and self-evaluation.^7^ Evidence in this regard, particularly in ALHIV in Africa and the SSA region, is limited.^17^ A Zambian study (2015) reported that ALHIV had few self-management skills to help them take antiretroviral treatment (ART) regularly.^18^

Self-management has been associated with better physical, psychological, knowledge and behavioural outcomes in people living with HIV.^19^ These outcomes have not yet been confirmed among ALHIV. A systematic review of the effectiveness of self-management interventions in youth with chronic conditions such as asthma, diabetes, HIV, cancer and cystic fibrosis found that self-management interventions that were focused on medical self-management and improved adherence to treatment.^20^ There is, however, little evidence of self-management interventions improving general coping with the chronic condition. Indeed, evidence from systematic reviews suggests that many self-management interventions do not have a sound theoretical basis^12,20^ and that HIV self-management has not been a research priority in SSA.^21^

The purpose of this article is to describe associations between adolescent HIV self-management and treatment adherence, viral suppression, sexual risk behaviour and HRQoL. The study was explorative in nature and is a secondary analysis of a larger study aimed to develop an instrument to measure adolescent HIV self-management.^22^ The theoretical hypotheses of associations between the construct of self-management and the proximal and distal outcomes as presented in the IFSMT are explored. We hypothesised that higher reported levels of self-management will be associated with treatment adherence, less risk-taking sexual behaviour, better HRQoL and better viral suppression rates.

## Methods

### Study population and design

This is a quantitative cross-sectional study of 385 ALHIV aged 13–18 years, from 11 healthcare facilities in the Western Cape, South Africa. Participants were required to complete a ‘self-report’ questionnaire. All healthcare facilities in the Cape Metropole with more than 50 adolescents on ART in care were canvassed. Adolescents who attended clinics for HIV care were recruited serially over a period of 5 months, from 13 March 2017 until 4 August 2017. Based on a previous study by Webel et al. that indicated a correlation between self-management and ART adherence as measured on a visual analogue scale, namely, *r* = 0.18, *p* < 0.01, a minimum sample size of 240 was required to provide 95% confidence interval.^23^ Participants were eligible if they knew their HIV status and had the capacity to complete the questionnaire. Of the participants approached, 27 either did not know their HIV status or parents informed the research team that their child was ‘slow’ and would not be able to comprehend the questions (Figure 1). No formal cognitive assessments were performed.

**FIGURE 1:**
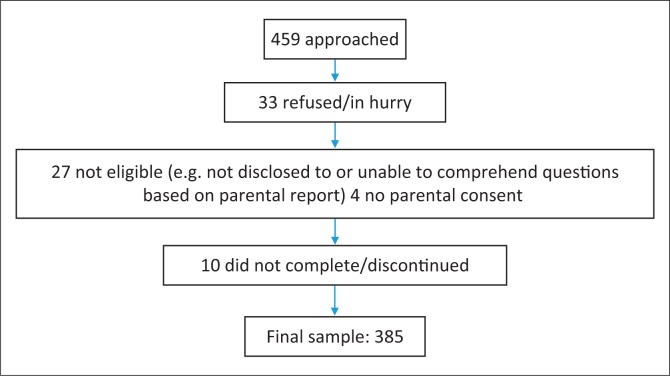
Study sample.

Data were collected by the researcher with the assistance of trained fieldworkers through paper-based self-report questionnaires. Participants either provided information on their own (70.4%, *n* = 271) or were assisted by fieldworkers (29.6%, *n* = 114). The questionnaires were available in the local languages (English, Afrikaans and isiXhosa) and were pretested with 33 participants prior to administration in the main study.

## Measures

### Demographic information

This section contained questions related to the individual, family and health background of the adolescent. Questions included gender, age, home language, highest grade completed and with whom the adolescent was residing. HIV-related information included how they became infected with HIV, when they were diagnosed with HIV, the age of disclosure, other health-related conditions (co-morbidities) and knowledge of their current CD4 count and viral load (VL) as a measure of their health literacy.^2^

### Self-management

The measure of self-management presented in this article was developed based on the processes of self-management as identified in the IFSMT. The developed 35-item measure of Adolescent HIV Self-Management (AdHIVSM-35) included five components of adolescent HIV self-management (Table 1),^24^ which was found to be a valid and reliable measure in this population.^22^ Items were measured with a four-point Likert scale. Two scale options were used: *strongly agree/always*; *agree/most of the time*; *disagree/sometimes* and *strongly disagree/never*. The minimum score for each item was 1 = poor self-management and the maximum score 4 = good self-management. The Cronbach’s alpha of the scale was 0.84 (subscales 0.55–0.76) and test–retest reliability 0.76.^22^

**TABLE 1:** Attributes of key components of adolescent HIV self-management.^22^

Component	Key attributes
Believing and knowing	Views or ideas about one’s illness, the future and confidence to self-manage. Awareness and comprehension of how to navigate the healthcare system and the importance of treatment (ART)
Goals and facilitation	Internal and external motivation for self-management by setting individual goals and by obtaining support from family, healthcare workers, peers and friends to take care of one’s health
Participation	Actively involved in own healthcare and in social pursuits
HIV biomedical management	Knowledge of and motivation to understand whether one is doing well on treatment or not. This includes knowledge of one’s viral load and names of ARVs
Coping and self-regulation	Manage HIV stigma, make decisions about disclosure and integrate taking treatment into one’s daily routine

*Source*: Based on Crowley T. The development of an instrument to measure adolescent HIV self-management in the context of the Western Cape, South Africa. [unpublished thesis]. Cape town: Stellenbosch University; 2018

ARVs, antiretroviral drugs; ART, antiretroviral treatment.

### Sexual risk behaviours

Sexual risk behaviour questions included whether participants ever had penetrative vaginal/anal sex, the frequency of sex (in the past 3 months), number of partners, the use of condoms, diagnosis of sexually transmitted infections and pregnancy. It is made up of 16 questions derived from the Youth Questionnaire for persons aged 15–24 years used in the Third South African National HIV, Behaviour and Health survey.^25^ The sexual risk behaviour questions did not include questions about sexual abuse, although this was asked in another part of the questionnaire. As the questionnaires were anonymous, the researchers could not take action on these responses if not explicitly reported by the participants.

For the structural equation model (SEM), sexual risk behaviour was calculated as follows: (1) if the response to the two questions, whether they ever had vaginal or anal sex, was ‘no’ in both cases, then the score = 0. (2) The response to the question on the number of sexual events in the last 3 months provided a score of 1–5. In cases where there was a ‘don’t know’ response, a score of 2 was assigned. (3) The responses on how often condoms were used were assigned the following scores: every time = 0, almost every time = 1, sometimes = 3 and never = 3; (4) Number of partners were scored 1–4. In conflicting cases where respondents indicated that they did have sex, but then responded with ‘not applicable’ to this question, the number of partners was assumed to be 1. (5) The final score was calculated by assigning a zero if case 1 above was applicable, or the sum of the numbers in cases 2, 3 and 4.

### Viral suppression

The most recent documented VL was obtained from the participant clinic folder. A VL of < 50 copies/mL was considered to be viral suppression.^26^ For the SEM model, the VL log value was used in the analysis.

### Adherence

Two Likert scale items were used. It included a rating of how often medication was missed over the past month and a rating of when was the last time the participant missed taking medication.^27,28^ The two items were dichotomised into adherent (indicating perfect adherence – never skipping or missing a dose) and non-adherent (reporting any missed dose).

*Non-adherent behaviour*: A list of reasons for non-adherence and the frequency thereof was taken from the Adult AIDS Clinical Trials Group (AACTG) Adherence questionnaire^29^ that was adapted for adolescents in 2004 by the Paediatric AIDS Clinical Trials Group.^30^ Response options included the following: never = 0; not often (1–2 times per month) = 1; sometimes (1–2 times per week) = 2 and often (more than 3 times per week) = 3. The total non-adherence score was calculated by adding the item codes for 0 = ‘never’ through 3 = ‘often’. The Cronbach’s alpha of this 17-item scale was 0.84.

In addition to the adherence questions, participants were asked how long they had taken ART, how many tablets they took each day and the frequency of daily doses. The current ART regimen was documented from the patient clinic folder.

### Health-related quality of life

Health-related quality of life was measured with the KIDSCREEN-27 which consists of 27 items and measures health and well-being on a five-point Likert scale.^14^ The KIDSCREEN-27 has five latent concepts: physical activities and health; general mood and feelings about yourself; family and free time; friends and school and learning. The Cronbach’s alpha coefficients of the KIDSCREEN-27 subscales range from 0.80 to 0.84 and test–retest reliability ranges from 0.61 to 0.74.^14^ In the present study, Cronbach’s alpha was 0.89 (subscales 0.74–0.82).

## Statistical analyses

Data were analysed with the Statistical Package for the Social Sciences (SPSS, version 25). Descriptive statistics included frequencies and percentages and means/medians and standard deviations (SDs)/interquartile ranges (IQRs). Bivariate Pearson’s correlation was used to test for an association between the total self-management score and the HRQoL and non-adherence behaviour scores. The independent *t*-test was used to establish mean differences in self-management scores across binary categories of adherence, viral suppression and sexual risk behaviour as self-management scores were normally distributed. For reliability of the instruments used in this study, Cronbach’s alphas were calculated and confirmatory factor analyses (CFAs) conducted using the R package Lavaan. Partial least squares structural equation modelling (PLS-SEM) using Smart PLS 3.2.6 was used to determine the relationships between self-management processes, proximal (non-adherence and sexual risk behaviour’s) and distal outcomes (HRQoL, VL). The model was created based on the IFSMT (Figure 2).

**FIGURE 2:**
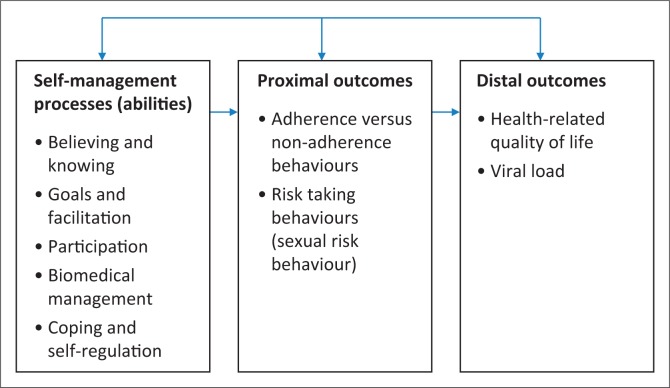
Conceptual framework based on the individual and family self-management theory.^7^

We tested for the direct influence of condition-specific, individual and environmental contextual factors on the self-management processes and outcomes in a separate SEM model. These factors included age, gender, adolescents’ knowledge of the route of infection, years on treatment, frequency of treatment and whether they were staying with a biological parent. Because the path coefficients and *p*-values did not significantly differ between the model where the covariates were included and the model without the covariates (see Appendix 1), only the model without the covariates is reported. A level of significance of <0.05 was used.

### Ethical consideration

Stellenbosch University Health Research Ethics Committee approval (Ref: S15/03/054) and Department of Health permission (Ref: WC_2015RP53_21) were obtained to conduct the study. Informed consent was obtained for all adult participants before data collection. Adolescent assent and parental consent (either in person or telephonically) were obtained for adolescents younger than 18.

## Results

### Reliability analysis

Reliability analyses were conducted on the measurement instruments that were used in this study, namely, self-management, non-adherence behaviour’s and quality of life. Cronbach’s alphas were calculated, and CFAs were conducted to determine whether this data set supported the latent structures of each of the instruments. In general, the reliability of the instruments was interpreted to be satisfactory (results not shown).

### Demographics

The sample included 58.2% (*n* = 224) females and 77.1% (*n* = 296) participants were isiXhosa-speaking. The median age was 15 and the IQR range was 14–16. More than a third (*n* = 138, 36.2%) had not completed the appropriate grade for their age. Participants most frequently reported residing with their biological mother (*n* = 151, 39.4%). The researcher determined the most likely route of infection based on information provided in the questionnaire, including age of diagnosis and sexual history. The majority of adolescents (*n* = 344, 89.4%) appeared to have been infected either perinatally or early in life (Table 2).^24^

**TABLE 2:** Demographics of the study sample.

Variable	*n*	%
**Age (calculated from date of birth) (*n* = 385)**		
13	73	19
14	74	19.2
15	65	16.9
16	74	19.2
17	57	14.8
18	42	10.9
**Home language (*n* = 384)**		
IsiXhosa	296	77.1
Afrikaans	53	13.8
English	25	6.5
Other	10	2.6
**Gender (*n* = 385)**		
Male	159	41.3
Female	224	58.2
I choose not to say	2	0.5
**Are you in school? (*n* = 383)**		
Yes	377	98.4
No	6	1.6
**Completed appropriate grade for age? (researcher determined) (*n* = 381)**		
Yes	243	63.8
No	138	36.2
**With whom do you stay? (*n* = 383)**		
Biological mother	151	39.4
Biological father	15	3.9
Biological mother and father	80	20.8
Family member (aunt, grandmother, sister, brother, etc.)	118	30.6
Adoptive parents	13	3.4
Other	6	1.6
**How long have you lived with this person? (*n* = 383)**		
Less than 1 year	31	8.1
1–5 years	37	9.7
6–10 years	37	9.7
More than 10 years	278	72.6
**Level of education of primary caregiver (*n* = 380)**		
No formal schooling	15	3.9
Primary school	57	15
High school	150	39.5
College or university	38	10
Not sure/don’t know	120	31.6
**Is your biological mother still alive? (*n* = 383)**		
Yes	267	69.7
No	106	27.7
Not sure	10	2.6
**Is your biological father still alive? (*n* = 379)**		
Yes	231	60.9
No	121	31.9
Not sure	27	7.1
**Are you still in contact with your biological mother and father? (*n* = 383)**		
Yes, with my mother and father	131	34.2
Yes, only with my mother	127	33.2
Yes, only with my father	40	10.4
No	85	22.2
**Number of people in the same house as you (*n* = 363)**		
Median (interquartile range)	4	3
**Number of times moved house in the past 5 years (*n* = 376)**		
Median (interquartile range)	1	2
**Nights stayed away from home in the past week (*n* = 375)**		
Median (interquartile range)	0	1
**When were you diagnosed with HIV? (*n* = 383)**		
At birth	192	50.1
Before the age of 6	33	8.6
Between 6 and 12	33	8.6
After the age of 12	56	14.6
Don’t know/not sure	69	18
**At what age did you find out you were HIV positive? (*n* = 375)**		
Between the ages of 6 and 10	159	42.4
Between the ages of 10 and 12	97	25.9
After the age of 12	119	31.7
**Do you have other conditions or illnesses? (*n* = 384)**		
Yes	52	13.5
No	309	80.5
I don’t know	23	6
**How did you become infected with HIV? (More than one option could be selected here therefore totals do not add to 100 % )**		
At birth/from my mother	282	73.2
By having sex	46	11.9
Forced sex or abuse	11	2.9
Shared needles or recreational drug equipment	8	2.1
Blood transfusion or other medical procedure	19	4.9
Don’t know	94	24.4
**Most likely route of infection (researcher determined)**		
Perinatally or early in life	344	89.4
Behaviourally	41	10.6

*Source:* Based on Crowley T. The development of an instrument to measure adolescent HIV self-management in the context of the Western Cape, South Africa. [unpublished thesis]. Cape town: Stellenbosch University; 2018

### Adherence

Only 44.8% (*n* = 168) and 38% (*n* = 143) of participants, respectively, reported that they never miss a dose of ART in the past month or never skipped their treatment. Most were on a first-line regimen (Table 3).^24^ The most frequently reported reasons for missing a dose of ART (*not often, sometimes or often*) was forgetting (*n* = 196, 52.7%) because they fell asleep or were still sleeping (*n* = 135, 36.2%) and that taking antiretroviral drugs (ARVs) reminded them of HIV (*n* = 124, 33.4%).

**TABLE 3:** Regimen, adherence and viral load.

Variable	*n*	%
**Current regimen (*n* = 377)**		
Abacavir (ABC), lamivudine (3TC) and efavirenz (EFV)	132	35
Tenofovir (TDF), emtricitabine (FTC) and EFV (fixed-dose-combination)	94	24.9
Zidovudine (AZT), 3TC and lopinavir/ritonavir (LPV/r)	52	13.8
ABC, 3TC and LPV/r	48	12.7
Other (seven participants or less per individual regimen)	51	13.6
**How often do you have to take your tablets? (*n* = 376)**		
Once a day	236	62.8
Twice a day	121	32.2
More than two times a day	14	3.7
Don’t know/not sure	5	1.3
**Viral load (*n* = 347)**		
Suppressed (< 50)	226	65.1
Not suppressed (> 50)	121	34.9
**When was the last time that you missed taking any of your ARVs? (*n* = 376)**		
Within the past week	117	31.1
1–2 weeks ago	52	13.8
2–4 weeks ago	14	3.7
1–3 months ago	20	5.3
More than 3 months ago	30	8
I never miss or skip	143	38
**In general, over the past month, how often did you miss taking your ARVs? (*n* = 375)**		
I hardly ever take any of my ARVs	5	1.3
I miss most of my ARVs	14	3.7
I miss about half of my ARVs	17	4.5
I miss my ARVs a little bit of the time	171	45.6
I never miss any of my ARVs	168	44.8

*Source:* Based on Crowley T. The development of an instrument to measure adolescent HIV self-management in the context of the Western Cape, South Africa. [unpublished thesis]. Cape town: Stellenbosch University; 2018

ARV, antiretroviral drugs.

### Antiretroviral drugs

The frequency of taking tablets was significantly associated with self-management scores (*F*[3.335] = 3.381, *p* = 0.02). Those who take tablets once daily had higher self-management scores compared to those who did not know or those who took more than once daily doses.

### Sexual risk behaviour

Almost a third (*n* = 121, 32%) of the participants in this study sample reported having penetrative vaginal sex, 26 (6.9%) penetrative anal sex and 45 (11.9%) oral sex. The mean age of sexual debut reported by 91 participants was 14.03 years (SD 2.14 and range 7–18); 38 participants indicated that they did not remember. Less than half of the participants used condoms every time they had sex (Table 4). Seventeen (12.9%) of the sexually active participants reported having a sexually transmitted infection in the past 3 months. Nine female adolescents (12.5% of sexually active females) reported being pregnant at the time of or before the completion of the questionnaire and nine male participants (15.5% of sexually active males) reported having made a female pregnant.

**TABLE 4:** Sexual risk behaviours.

Variable	*n*	%
**In the past 3 months, how many times you had penetrative vaginal or anal sex? (*n* = 130)**		
0	31	23.8
1	20	15.4
2	26	20
3	17	13.8
More than 5	18	13.8
Don’t know	18	13.8
**Of those times in the past 3 months that you had sex, how many times did you use a condom? (*n* = 129)**		
Never	20	15.5
Sometimes	20	15.5
Almost every time	17	13.2
Every time	52	40.3
Don’t know	6	4.7
Not applicable	14	10.9
**In the past 3 months, how many different partners did you have vaginal or anal sex with? (*n* = 130)**		
1	59	45.4
2	18	13.8
3	7	5.4
More than 3	5	3.8
Don’t know	14	10.8
Not applicable	27	20.8
**Did you or your partner use anything to keep from getting pregnant the last time you had vaginal sex? (*n* = 129)**		
Yes	83	64.3
No	32	24.8
I can’t remember	14	10.9

*Source:* Based on Crowley T. The development of an instrument to measure adolescent HIV self-management in the context of the Western Cape, South Africa. [unpublished thesis]. Cape town: Stellenbosch University; 2018

Of the 385 participants, 25 (*n* = 6.5%) reported sexual abuse within the past 3 months. Of these 25, 17 were female and 8 were male; 16 were in the age category of 16–18 years and 9 were in the age category of 13–15 years.

### Health-related quality of life

Health-related quality of life is a subjective measure of one’s own health and well-being. The majority (*n* = 354, 92.4%) of participants reported excellent, very good or good overall health (Table 5). Most participants reported very good or excellent levels of *Physical activities and health*. On the *Mood and feelings* scale, most participants reported that, for example, they very often or always enjoyed their life. The scores for the above-mentioned sub-scales were in the same range as international norms, indicating that the participants’ HRQoL was similar to other population groups. Means for the *Family and Free Time* and *Friends* sub-scales were slightly lower compared to international norms and the mean for *School and Learning* higher. Lower scores on the *Family and Free Time* sub-scale seemed to be related to lower ratings with regard to the availability of money. Most participants reported that they were happy at school.

**TABLE 5:** Health-related quality of life.

HRQoL scores	*n*	Alpha	Mean	SD	Median	IQR	Rasch person parameters mean	SD	*T* mean	SD	European norms	SD
Total HRQoL score	328	0.893	102.2	17.6	105	24	-	-	-	-	-	-
Physical activities and health	373	0.754	17.3	4.6	18	7	0.78	1.7	46.98	12.1	46.83	9.2
Mood and feelings	368	0.735	27.6	5.4	28.5	8	1.5	1.5	48.56	11.4	47.3	9.6
Family and free time	365	0.816	24.9	6.6	25	10	0.74	1.3	45.79	12.6	48.53	9.8
Friends	378	0.773	14.6	4.2	16	6	1.12	1.9	45.63	12.7	50.07	9.9
School and learning	371	0.773	16.1	3.4	17	5	1.89	1.8	53.87	11.3	48.54	9.2

*Source:* Based on Crowley T. The development of an instrument to measure adolescent HIV self-management in the context of the Western Cape, South Africa. [unpublished thesis]. Cape town: Stellenbosch University; 2018

HRQoL, health-related quality of life; IQR, interquartile ranges; SD, standard deviation.

### Self-management

Participants generally had high self-management ratings. Self-management items that participants seemed to struggle with (items with mean scores below 3) were coping with HIV stigma, participating in healthcare, communicating with healthcare providers about missing treatment or private issues, participating or finding help in the community, knowing the names of one’s ARVs or one’s VL, showing interest in understanding one’s VL and remembering to take treatment (not relying on other people to remind them). The sub-scales with the lowest mean percentage scores were *Biomedical management* and *Coping and self-regulation* (Table 6). Participants who indicated that they did not know how they were infected had significantly lower self-management scores compared to those who knew (*t*[115.15] = −2.299, *p* = 0.02).

**TABLE 6:** Self-management sub-scale percentage scores.

Self-management	*n*	Alpha	Mean	SD	Min	Max
**Total AdHIVSM-35**	340	0.839	79.6	7.4	41	99
1. Believing and knowing	369	0.761	89.4	10.7	38	100
2. Goals and facilitation	378	0.708	87.8	12.4	44	100
3. Participation	372	0.715	73.7	14.4	33	100
4. Biomedical management	374	0.651	69.7	17.5	25	100
5. Coping and self-regulation	360	0.547	68.6	17.1	25	100

*Source:* Based on Crowley T. The development of an instrument to measure adolescent HIV self-management in the context of the Western Cape, South Africa. [unpublished thesis]. Cape town: Stellenbosch University; 2018

AdHIVSM-35, Adolescent HIV Self-Management 35-item scale; standard deviation.

Numbers 1–5 indicate the sub-scales of the AdHIVSM-35. For explanation of the sub-scales, refer to Table 1.

Adolescent HIV self-management had a correlation coefficient of medium strength with HRQoL (*r* = 0.450, *p* < 0.01) and a negative correlation with non-adherent behaviour (*r* = −0.249, *p* < 0.01). The sub-scale of self-management with the strongest correlation with HRQoL was *Goals and facilitation*, which includes setting goals, but importantly, obtaining support from family, friends and healthcare workers. *Participation* or being actively involved in one’s care and in social pursuits was the sub-scale that had the strongest negative correlation with non-adherent behaviour.

Adolescents who reported higher HIV self-management were more likely to be adherent to treatment (*t* = 4.435 [336], *p* < 0.01), virally suppressed (*t* = 2.376 [305], *p* = 0.02) and practise consistent condom use (*t* = 1.947 [95], *p* = 0.05) (Table 7).

**TABLE 7:** Independent *t*-tests for adolescent HIV self-management across categories of viral suppression and adherence.

AdHIVSM-35	*n*	Mean	SD	*T*	DF	*p*
VL suppressed < 50						
Yes	197	80.56	9.52	2.376	305	0.02
No	110	77.90	9.22	-	-	-
Adherent (Likert item 1 – last missed dose)						
Yes	130	82.43	9.04	4.435	336	<0.001
No	208	77.91	9.19	-	-	-
Adherent (Likert item 2 – average adherence)						
Yes	160	81.97	9.21	4.444	336	<0.001
No	178	77.54	9.08	-	-	-
Consistent condom use						
Yes, using condoms every time	46	81.15	8.59	1.947	95	0.05
No, inconsistent condom use	51	77.58	9.38	-	-	-
Multiple sexual partners						
Yes, more than one partner	27	77.86	9.73	−1.187	78	0.24
One partner only	53	80.44	8.95	-	-	-

*Source:* Based on Crowley T. The development of an instrument to measure adolescent HIV self-management in the context of the Western Cape, South Africa. [unpublished thesis]. Cape town: Stellenbosch University; 2018

AdHIVSM, adolescent HIV self-management; SD, standard deviation; DF, degrees of freedom.

### Structural equation modelling model

**FIGURE 3:**
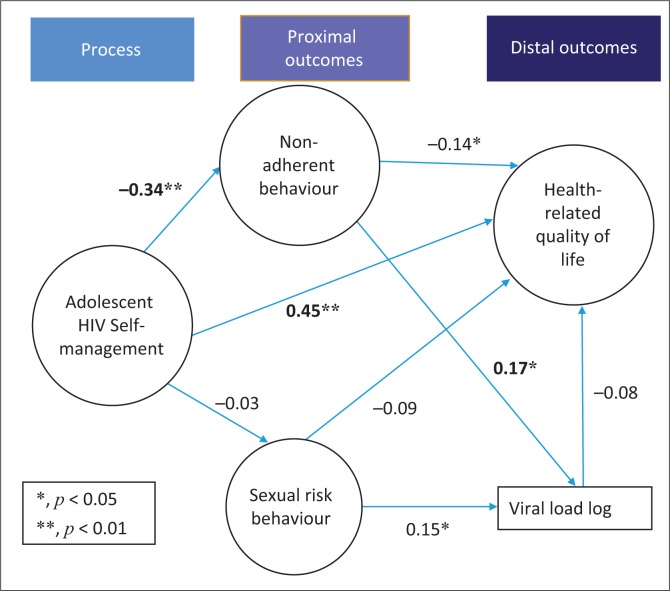
Partial least squares structural model.

Figure 3 shows the PLS structural model indicating a significant relationship between self-management and HRQoL (*r* = 0.45, *p* < 0.01). Non-adherent behaviour appears to mediate the relationship between self-management and viral suppression. As shown in Figure 3, non-adherent behaviour was negatively correlated with self-management (*r* = −0.34, *p* < 0.01) and positively correlated with VL log (*r* = 0.17, *p* < 0.05). This means that lower self-management is associated with more non-adherent behaviour which, in turn, influences VL levels. Non-adherent behaviours had a moderate negative association with HRQoL. There also appears to be a positive correlation between sexual risk behaviour and the VL level (*r* = 0.15, *p* < 0.05).

The relationship between self-management and sexual behaviour was not significant.

## Discussion

The current study explored relationships between variables based on a framework developed from the IFSMT and therefore cause and effect relationships cannot be inferred. However, the findings of this study support the theory and previous systematic reviews that higher self-management may influence treatment adherence, certain health behaviours, HRQoL and treatment outcome.^12,19,20,21^ The limitation identified in systematic reviews has been that most self-management interventions had no theoretical basis.^12,20^ The findings of this study may indicate that interventions that have a comprehensive focus, that include components to address the various self-management processes, may affect both the medical management, for example, adherence and the psychosocial outcomes, such as HRQoL.

The study also yielded some descriptive data with regard to self-management processes and the proximal (adherence and sexual risk behaviour) and distal outcomes (HRQoL and viral suppression). Self-management aspects that participants found challenging concerned knowledge of their treatment, for example, names of their ARVs and an understanding of whether they are doing well on treatment or not. It was challenging to manage HIV stigma, make decisions about disclosure and integrate taking treatment into their daily routine.

Less than half of the participants reported complete adherence in the two Likert scale items in this study. Low adherence rates amongst ALHIV have also been reported in other studies.^3,28,31^ This study supports the theory that low adherence rates are a concern and explains why adherence is a consistent component of self-management interventions for people living with HIV.^13^ Although self-management interventions that focus on adherence have been shown to improve treatment taking behaviour,^20^ self-management interventions must meet a broad range of needs.^13,17^ New interventions to address psychosocial support and mental health needs of ALHIV are needed. Currently, no single adherence strategy has been identified that improves adherence amongst ALHIV.^11^

Viral suppression rates (65.1%) in the present study were similar to other adolescent studies, namely, 32.5% – 76%.^3,32,33^ Other studies reported non-adherence between 30% and 45%^31,32,33^ whereas in our study it was between 55% and 62%. This may be because of differences in the measurement of non-adherence and the limitation of the current study that the VL was obtained from routine clinic records and not collected at the same time as questionnaires. We found that non-adherent behaviour mediates the relationship between self-management and the lack of viral suppression which is consistent with the IFSMT. Although biological markers have been the outcomes for some self-management interventions, VLs may be specifically related to medication self-management, which is only one component of chronic illness self-management. Self-management interventions may lead to improvement in the management of symptoms, coping, communication, participation and social roles without an effect on biological measures. Researchers should consider including outcomes such as quality of life or other psychological measures to measure the effect of self-management interventions whilst not excluding biological measures.^13^

In this study, almost a third of the participants reported having sex. The percentage is higher than in other studies amongst perinatally infected adolescents in Thailand, the United States/Porto Rico and South Africa.^32,33,34^ The present study included perinatally infected and behaviourally infected adolescents, which may be the reason for higher reported sexual activity. Sadly, a number of participants also reported sexual abuse, emphasising that clinicians should explicitly ask about sexual abuse during history taking. Further research is needed to explore sexuality and sexual risk behaviours amongst ALHIV. A study conducted in Botswana found that parents’ inaccurate perception of their adolescent’s sexual relationships was significantly associated with more risk-taking behaviours, emphasising the importance of parent–adolescent communication.^35^ Our study only found a borderline significant association between self-management and consistent condom use; according to the IFSMT, higher levels of self-management is associated with better health behaviour.^7^ Modelling in our study did not indicate a significant association between self-management and sexual behaviour. This may also be because the self-management scale used (AdHIVSM-35) did not specifically focus on sexual behaviour. Future studies should focus on developing instruments specifically for self-management of sexual behaviour.

Bernardin et al. (2013) recommended a culturally appropriate quality of life measurement as a key outcome for self-management interventions.^13^ Currently, there are no reference norms for HRQoL as measured by KIDSCREEN-27 amongst adolescents in South Africa. All the sub-scale mean scores were in the international range of 45–55, with SDs close to the international range of 10.^36^ This may indicate the subjective nature of HRQoL as well as the resilience of ALHIV. Nöstlinger et al. (2015) used the Family and Free Time (parents and home life) and Friends (social support by peers) sub-scales in their study in Kampala, Uganda and Western Kenya and reported mean values of 24 (SD 5.7) and 15.6 (SD 6.2), respectively, for the sub-scales, which is comparable to the mean values found in the present study.^37^ We found that there was a moderately significant relationship between self-management and HRQoL that is consistent with the IFSMT. However, evidence from systematic reviews suggests no clear effects of self-management interventions with regard to the HRQoL of young people living with chronic conditions^20^ or people living with HIV.^19^ More research is needed to explore this relationship.

## Limitations

The limitations of this study include the cross-sectional nature thereof, the reliance on self-report, specifically with regard to adherence and sexual risk behaviours, and the use of documented VLs. We did not assess cognitive function in this study. Cognitive delay may be an important domain to assess and further research with regard to the relationship between cognitive functioning and self-management is needed. Although more than a third of the participants were not in the correct grade for their age, other factors such as missing school because of ill health or attending appointments may also influence educational delay.^38^ The timeframe between the last VL measure and completion of the self-report questionnaire was not recorded. Although correlation coefficients were not strong, it is similar to what is reported in other studies.^23^

## Conclusion

Targeting adolescents’ skills related to HIV self-management in the clinical setting may improve adolescents’ adherence to treatment, viral suppression rates and their HRQoL. The relationship between self-management and sexual risk behaviour needs to be explored further. Sustainable self-management programmes for adolescents in primary healthcare settings should be developed and tested. Caregivers and healthcare workers can be involved and trained to support adolescents with self-management.

## Figures and Tables

**FIGURE 1-A1:**
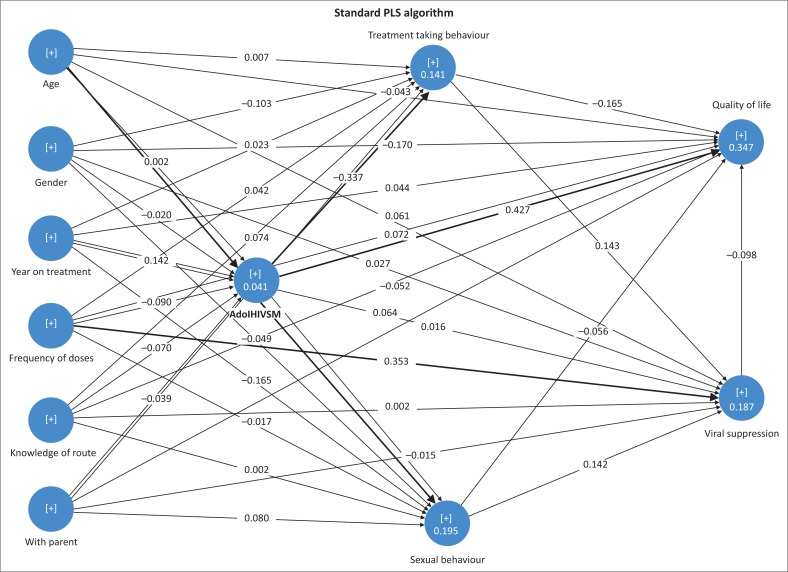
Model with all control paths.

**FIGURE 2-A1:**
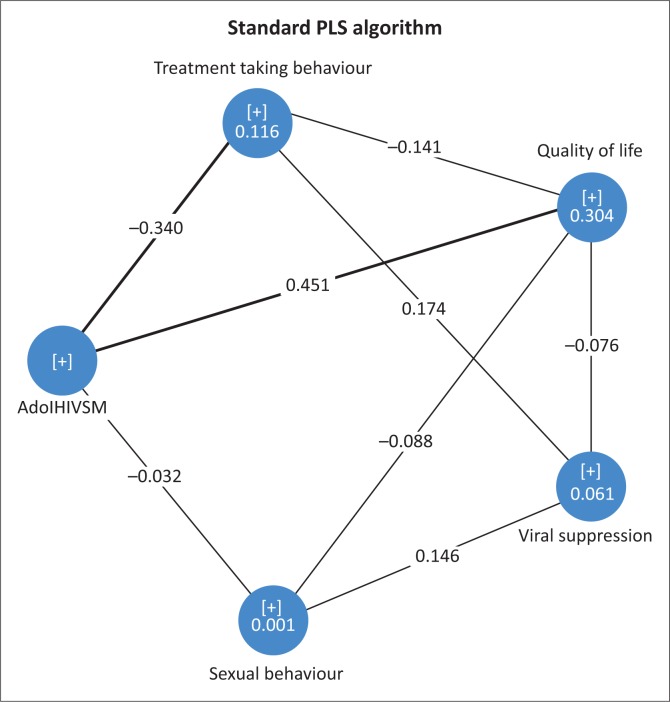
Model without controls.
